# A combination of selected mapping and clipping to increase energy efficiency of OFDM systems

**DOI:** 10.1371/journal.pone.0185965

**Published:** 2017-10-12

**Authors:** Byung Moo Lee, You Seung Rim, Wonjong Noh

**Affiliations:** 1 School of Intelligent Mechatronics Engineering, Sejong University, Seoul 05006, Korea; 2 Samsung Electronics Co., Ltd., Suwon 16677, Korea; Universita degli Studi della Tuscia, ITALY

## Abstract

We propose an energy efficient combination design for OFDM systems based on selected mapping (SLM) and clipping peak-to-average power ratio (PAPR) reduction techniques, and show the related energy efficiency (EE) performance analysis. The combination of two different PAPR reduction techniques can provide a significant benefit in increasing EE, because it can take advantages of both techniques. For the combination, we choose the clipping and SLM techniques, since the former technique is quite simple and effective, and the latter technique does not cause any signal distortion. We provide the structure and the systematic operating method, and show the various analyzes to derive the EE gain based on the combined technique. Our analysis show that the combined technique increases the EE by 69% compared to no PAPR reduction, and by 19.34% compared to only using SLM technique.

## Introduction

Increasing energy efficiency (EE) is a vital research topic in the current information technology (IT) field for the reduction of system operational cost and environmental pollution [1]. Orthogonal Frequency Division Multiplexing (OFDM) is a powerful signal transmission technique which is used for the most of the high data wireless signal transmission systems, and it is already adopted various standards including Advanced Television Systems Committee (ASTC) 3.0, 3rd Generation Partnership Project Long Term Evolution (3GPP LTE)-A/Pro, IEEE 802.11 n/ax, and so on [2, 3]. One of the main problems posed by the OFDM is its high peak-to-average power ratio (PAPR) which seriously limits the energy efficiency of the signal transmission systems. This is due to the fact that, to transmit a high PAPR signal such as OFDM, the input back-off (IBO) of the signal must be set high before the OFDM signal passes through the power amplifier (PA) to reduce both in-band distortion and out-of-band radiation. The PA is the most power hungry device, and reducing the power consumption of the PA is quite important to increase the EE of the OFDM system. By placing a digital predistorter (DPD) in front of the PA, the linear dynamic range of the PA can be increased to a certain range [4–7]. However, the DPD is an expensive device and the increment of the energy efficiency is rather limited. For this reason, PAPR reduction techniques for increasing EE are also widely used. Since this problem is very important from a practical system perspective, many studies have been conducted so far [8–12]. There are roughly two types of PAPR reduction techniques, distortion PAPR reduction techniques and distortionless PAPR reduction techniques. The distortion PAPR reduction technique is a method of reducing the PAPR of the OFDM signal by distorting the signal. Usually this kind of technique is simple and effective but involves signal distortion. Clipping is one of the most common distortion PAPR reduction techniques. The distortionless PAPR reduction technique reduces the PAPR of an OFDM signal without signal distortion. Usually this kind of technique can increase EE without signal distortion, but high computational complexity and delay are accompanied. Combining the distortion and distortionless PAPR reduction techniques, and choosing the appropriate parameters from there, are expected to increase EE more effectively.

In this paper, we show how to systematically combine the distortion and distortionless PAPR reduction technique. For the distortion PAPR reduction technique, we choose clipping technique since it is the most common and widely used PAPR reduction technique. For the distortionless PAPR reduction technique, we choose selected mapping (SLM) technique, since it needs minimum or no additional resources that most of distortionless PAPR reduction technique requires. The combination could enhance the advantages and compensate the disadvantages of each techniques. We propose a structure and systematic operation of the combined technique, and show how much of EE gain can be expected based on analysis and numerical simulations using the proposed technique. Based on our analysis, it can be seen that the combined technique provide very high EE gain and can be applied to most of OFDM based signal transmission system. There are several works related to the combination of SLM and clipping [13–15]. The previous works has more focused on introducing the method and performance analysis, while our work more focuses on systematic design and related operations. We show how the combined method can work in practical situation in detail.

In what follows, in Section 2, we present a system description for the study of this paper. We show the definition of PAPR and introduce each PAPR reduction technique, clipping and SLM. The definition of EE is also introduced for use as a metric in performance analysis. In Section 3, we propose the combined technique and related apparatus, and show the systematic operation. Section 3 includes most of design process and methodology. In Section 4, numerical analysis is shown to validate the proposed technique, and conclusion remarks are given in Section 5.

## System description

This section shows the definition of PAPR for OFDM and then shows clipping and SLM PAPR reduction schemes. The EE metric is also introduced to use in performance analysis.

### PAPR of OFDM signal

An OFDM signal of *N* subcarriers can be represented as

$$\begin{array}{c} x\left(t\right)=\frac{1}{\sqrt{N}}\sum_{k=0}^{N-1}X\left[k\right]e^{j2\pi f_{k}t},\;\;0\leq t\leq T_{s} \end{array}$$ (1)

where *T*_*s*_ is the duration of the OFDM signal and $f_{k}=\frac{k}{T_{s}}$.

The high PAPR of the OFDM signal arises from the summation in the above IDFT expression. The PAPR of the OFDM signal in the analog domain can be represented as

$$\begin{array}{c} PAPR_{c}=\frac{{\text{max}}_{0\leq t\leq T_{s}}{\left|x\left(t\right)\right|}^{2}}{E\left({\left|x\left(t\right)\right|}^{2}\right)} \end{array}$$ (2)

Nonlinear distortion in PA occurs in the analog domain, but most of the signal processing for PAPR reduction is performed in the digital domain. The PAPR of digital domain is not necessarily the same as the PAPR in the analog domain. However, in some literature [16, 17], it is shown that one can closely approximate the PAPR in the analog domain by oversampling the signal in the digital domain. Usually, an oversampling factor *L* = 4 is sufficient to satisfactorily approximate the PAPR in the analog domain, and it is also used in this paper.

The oversampled OFDM signal can be represented as

$$\begin{array}{c} x\left(n\right)=\frac{1}{\sqrt{LN}}\sum_{k=0}^{LN-1}X\left[k\right]e^{j2\pi nk/LN},\;\;0\leq n\leq LN, \end{array}$$ (3)

Then, we can express PAPR of the OFDM signal as follows.

$$\begin{array}{c} PAPR=\frac{{\text{max}}_{0\leq n\leq LN}{\left|x\left(n\right)\right|}^{2}}{E\left({\left|x\left(n\right)\right|}^{2}\right)}. \end{array}$$ (4)

To measure the PAPR performance, we usually use complementary cumulative distribution function (CCDF) and the CCDF of the Nyquist sampling rate is shown as follows:

$$\begin{array}{c} Pr\left(PAPR>PAPR_{0}\right)\approx 1-{\left(1-e^{-PAPR_{0}}\right)}^{N}. \end{array}$$ (5)

where *Pr*(⋅) represents the probablity, *PAPR*_0_ is the reference PAPR, and *N* is the number of subcarriers. Eq (5) can be derived on the assumption that the power of OFDM signal can be approximated by a central chi-square distribution with two degrees of freedom, and the OFDM samples are independent of each other, which is true in the case of a Nyquist sampling rate.

However, (5) is not so useful from a practical system point of view, because it cannot capture the actual PAPR of continuous time OFDM signal. For this reason, many continous time PAPR of OFDM CCDF models have been proposed. In [18], authors modified the Eq (5), and proposed continuous time PAPR using heuristic approach as:

$$\begin{array}{c} Pr\left(PAPR>PAPR_{0}\right)\approx 1-{\left(1-e^{-PAPR_{0}}\right)}^{\alpha N}. \end{array}$$ (6)

where *α* is the arbitrary adjustable parameter and it is known that *α* = 2.8 is a good choice for the approximation.

In [19], authors presented more analytical approximation which can be represented as:

$$\begin{array}{c} Pr\left(PAPR>PAPR_{0}\right)\approx 1-\exp\left(-\sqrt{\frac{\pi \cdot PAPR_{0}}{3}}Ne_{}^{-PAPR_{0}}\right). \end{array}$$ (7)

And another analytical approach was presented in [20] as:

$$\begin{array}{c} Pr\left(PAPR>PAPR_{0}\right)\approx 1-\exp\left(-\sqrt{\frac{\pi \cdot \ln N}{3}}Ne_{}^{-PAPR_{0}}\right). \end{array}$$ (8)

All of three approximations are well-matched with the CCDF of continuous time or *L* > 4 times oversampled OFDM signal.

### PAPR reduction techniques

In this subsection, we introduce each PAPR reduction technique, clipping and SLM, and present the characteristics of the techniques. The analysis of this subsection can be useful for the better understanding of the combined technique. In this paper, we use 10*MHz* bandwidth with 1024 subcarriers for the OFDM signal.

#### Clipping

Due to its simplicity and effectiveness, clipping is the most widely used as a PAPR reduction technique for the OFDM signal [21]. However, clipping causes both in-band distortion and out-of-band radiation. Setting the clipping ratio high enough can negate the in-band distortion. If the clipping ratio is relatively low, one usually recovers the performance degradation from in-band distortion using channel coding [22]. The reconstruction technique of the clipped OFDM signal by adding additional complexity at the receiver has been also proposed [23].

The output signal of clipping procedure, $\hat{x}\left(n\right)$ can be expressed as follows:

$$\begin{array}{c} \hat{x}\left(n\right)=\{\begin{array}{cc} x(n), & \left|x\left(n\right)\right|\leq A_{max}\\ A_{max}e^{j\theta_{n}}, & |x\left(n\right)|>A_{max}, \end{array} \end{array}$$ (9)

where *x*(*n*) is the OFDM signal before clipping procedure, *A*_*max*_ is the maximum allowable signal amplitude and *θ*_*n*_ is the phase of OFDM signal. The clipping level can be measured by using clipping ratio (CR), *ν* which is expressed as follows [7]:

$$\begin{array}{c} \nu =\frac{A_{max}}{\sqrt{P_{ave}}}, \end{array}$$ (10)

where *P*_*ave*_ is the average input power of OFDM signal.

Due to serious out-of-band radiation, filtering should be followed by clipping. As a filtering technique, we use the Frequency Domain Filtering (FDF) which was proposed in [17].

**Fig 1:**
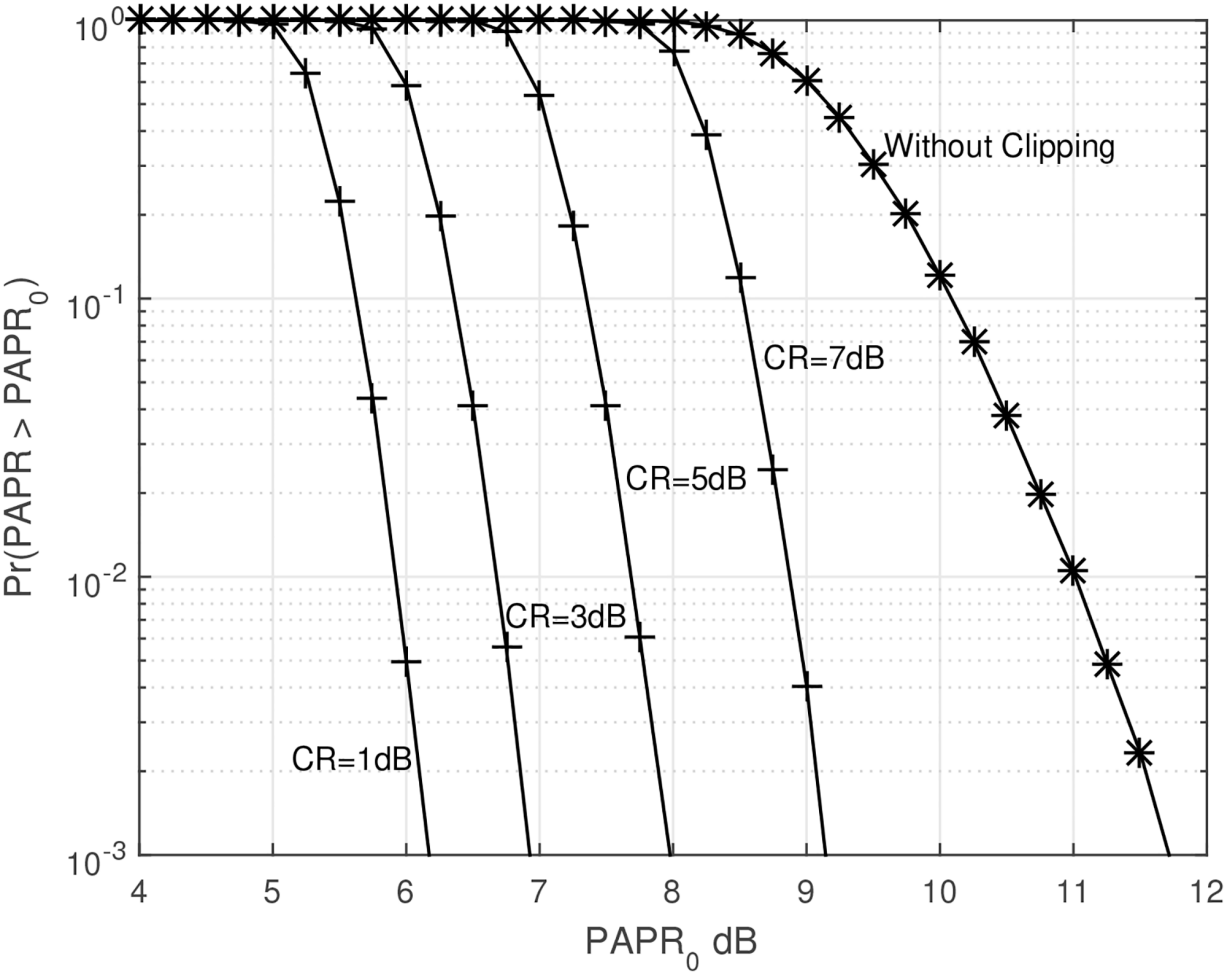
CCDF of clipping PAPR reduction technique.

**Fig 2:**
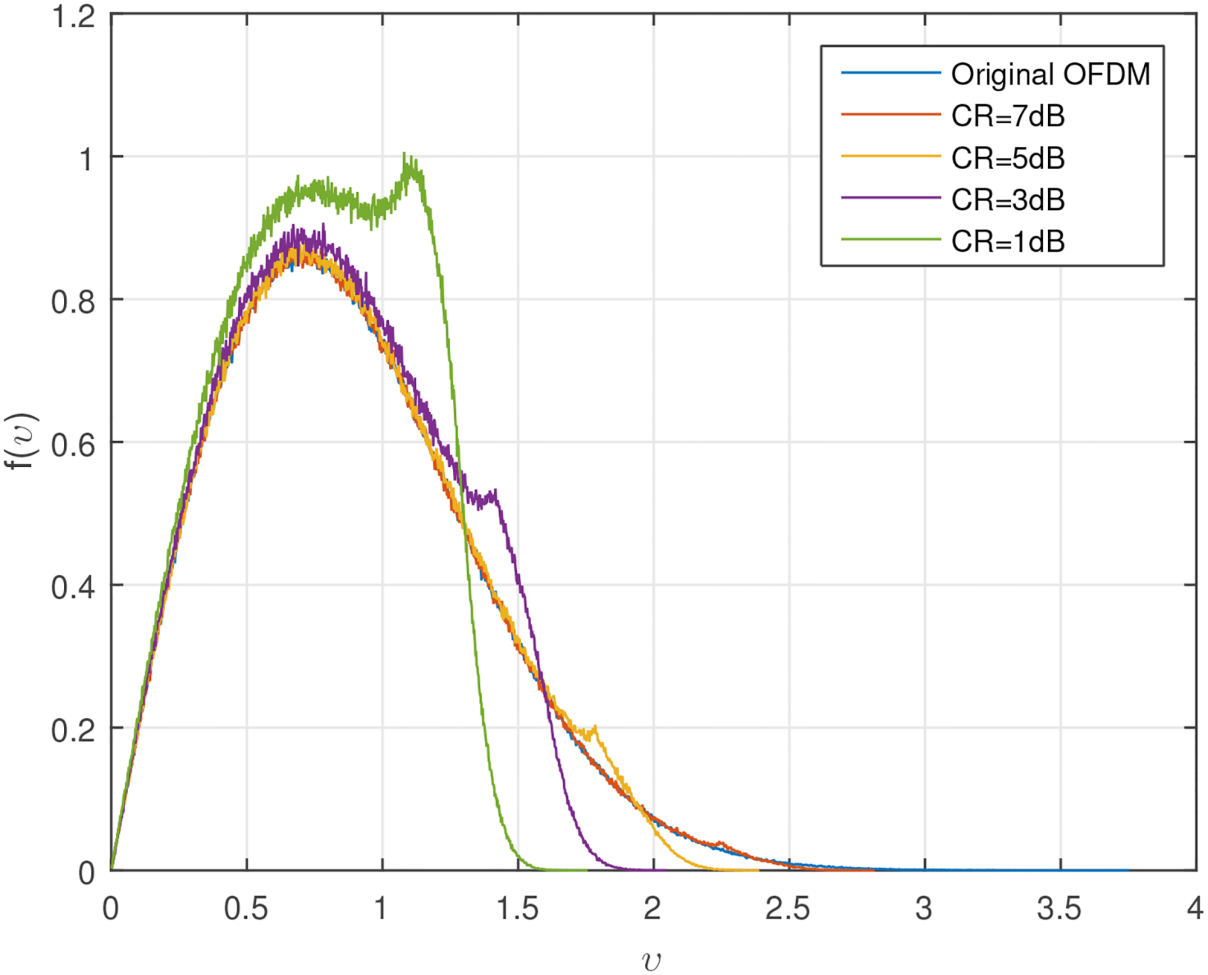
PDF of clipped OFDM signal amplitude.

Figs 1 and 2 show the CCDF performance and probability density function (PDF) of clipping PAPR reduction technique. From Fig 1, it is obvious that if we decrease the CR, the CCDF performance becomes better, while we must bear the clipping distortion. Also, we can see from Fig 2, the amplitude distribution of the original OFDM signal has a Rayleigh distribution, but the amplitude distribution of the clipped OFDM signal becomes closer to a uniform distribution as clipping amount increases. The tremendous peak-regrowth after filtering can be reduced if we combine the CAF (clipping and filtering) techniques with the SLM technique which will be shown soon.

The clipping PAPR reduction technique is a kind of distortion PAPR reduction technique which means reduce the PAPR of the signal based on signal distortion, so a relevant level of distortion is inevitable. The clipping PAPR reduction technique is a nonlinear function and it is well-known in the literature that, for the linear receiver, any kind of nonlinear function can be represented based on Bussgang Theorem [24]:

$$\begin{array}{c} \hat{x}\left(n\right)=\alpha x\left(n\right)+d\left(n\right), \end{array}$$ (11)

where *d*(*n*) is the distortion noise term due to clipping which is not related to *x*(*n*), and *α* is the attenuation term due to clipping which can be represented as [22]:

$$\begin{array}{ccc} \alpha & = & \frac{E\left[x{\left(n\right)}^{*}\hat{x}\left(n\right)\right]}{E\left[{\left|x\left(n\right)\right|}^{2}\right]}\\ & = & 1-e^{-{\left(\nu \right)}^{2}}+\frac{\sqrt{\pi }\nu }{2}erfc\left(\nu \right) \end{array}$$ (12)

where *E*[⋅] is the expectation operation, * is the complex conjugate operation, *ν* is the clipping ratio. For (12), we assumed there is no correlation between distortion noise term and signal term (*E*(*x*(*n*)*d(*n*)) = 0).

The frequency representation of the clipping noise term *d*(*n*) can be written as

$$\begin{array}{c} D\left[k\right]=\frac{1}{\sqrt{N}}\sum_{n=0}^{N-1}d\left(n\right)e^{-j2\pi nk/N}. \end{array}$$ (13)

As the number of subcarriers increases, *D*[*k*] approaches a complex Gaussian random variable with a zero mean due to the central limit theorem. According to Parseval’s theorem, the expectation of the |*D*[*k*]|^2^, *E*(|*D*[*k*]|^2^) also can be calculated in the time domain. From (9), the amplitude of distortion noise |*d*(*n*)| can be given by,

$$\begin{array}{c} \left|d\left(n\right)\right|=|x(n)|-A_{\text{max}},\;\;\;\;\;\;|x(n)|>A_{\text{max}}, \end{array}$$ (14)

Since it is well-known that the amplitude of the OFDM signal, |*x*(*n*)| has a Rayleigh distribution, the average distortion power, *E*(|*d*(*n*)|^2^) can be expressed as follows:

$$\begin{array}{c} E\left({\left|d\left(n\right)\right|}^{2}\right)=\int_{A_{\text{max}}}^{\infty }{\left(r-A_{\text{max}}\right)}^{2}p_{\left|x\left(n\right)\right|}\left(r\right)dr, \end{array}$$ (15)

where *p*_|*x*(*n*)|_(*r*) is the Rayleigh probability density function (pdf). Then, with normalized signal power assumption, (15) can be simplified as [25],

$$\begin{array}{c} E\left({\left|d\left(n\right)\right|}^{2}\right)=e^{-\nu^{2}}-\sqrt{\pi }\nu \cdot erfc\left(\nu \right). \end{array}$$ (16)

In the practical systems, due to the peak-regrowth, actual clipping ratio after filtering is usually much higher than clipping ratio we set. For this reason, the analysis of this subsection does not directly applied to the real system, but should be modified based on adjustable factor. We will show this in the following section.

#### Selected mapping

**Fig 3:**
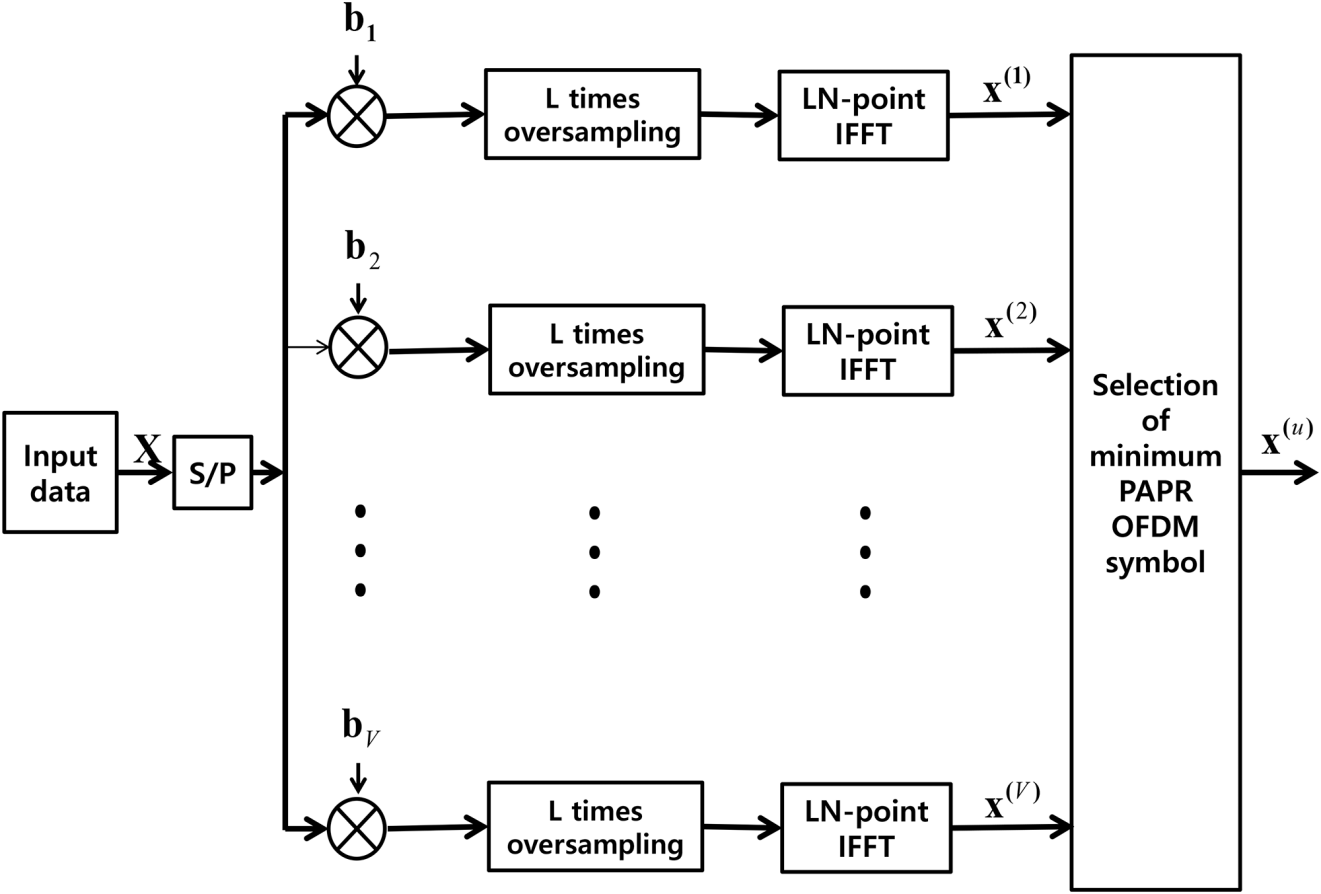
Block diagram of SLM.

The SLM technique is a kind of distrotionless PAPR reduction technique, and it reduces the PAPR of OFDM symbol by rotating phase of the OFDM symbol. The SLM technique makes several candidate phase sets from origianl OFDM symbol based on phase rotation, and choose the one which shows the best PAPR performance. There are two representative phase rotation PAPR reduction techniques, SLM [26] and partial transmission sequences (PTS) [27]. Between them, we choose SLM, since both technique shows very much similar characteristics, but SLM is easier to apply. The block diagram of SLM is shown in Fig 3. The input data **X** is multiplied with various phase sets **b_i_**, *i* = 1, 2, ⋯, *V*, in frequency domain and generate *V* different time domain symbols, **x**^(*i*)^, *i* = 1, 2, ⋯, *V*. Then, among *V* different symbols, the lowest PAPR symbol is chosen and send it to the receiver through the channel. Side Information (SI) is necessary to decode the signal block since the receiver must know which phase set was chosen at the transmitter. Because of the high priority of the SI, it is usually heavily protected by channel coding [22]. Several blind techniques with additional complexity were also proposed [28, 29].

**Fig 4:**
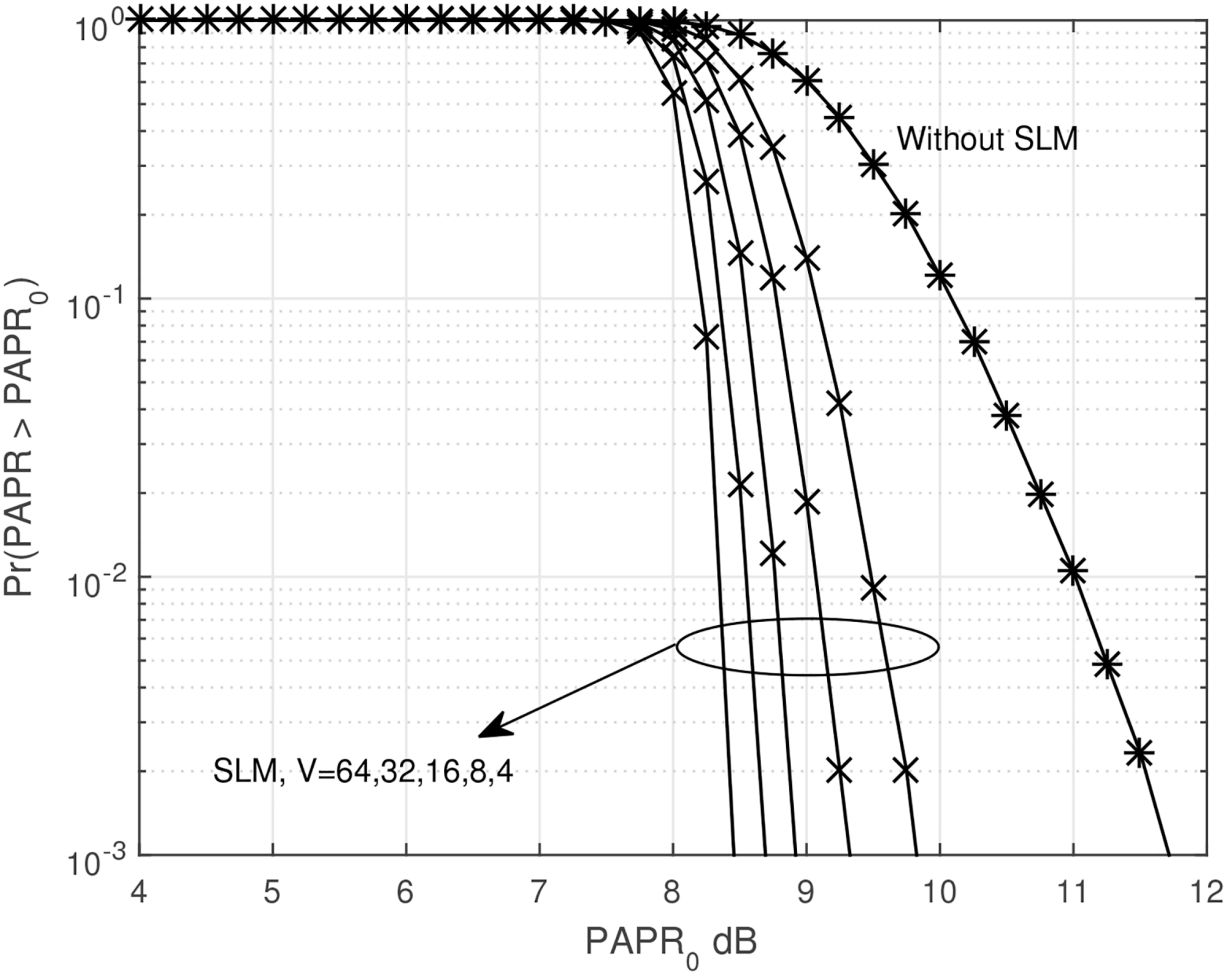
CCDF of OFDM signal with SLM technique.

**Fig 5:**
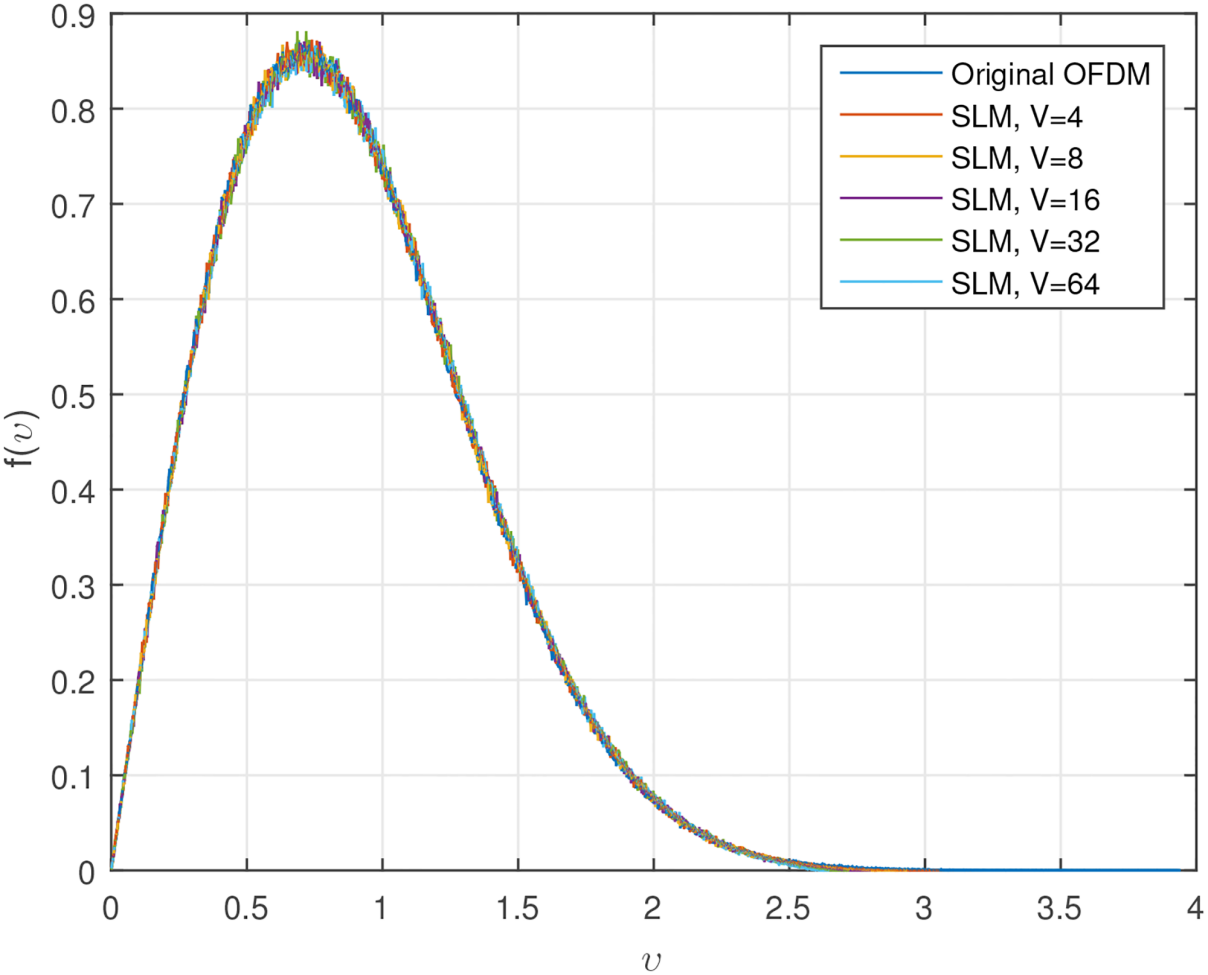
PDF of OFDM signal amplitude with SLM technique.

Figs 4 and 5 show the CCDF of OFDM signal with SLM technique, and PDF of OFDM signal amplitude with SLM technique.

As we can see from Figs 4 and 5, SLM technique can successfully reduce the PAPR of OFDM signal without any signal distortion. However, the complexity due to the generation of multiple signal sets could be a problem. It is well-known in the literature that the CCDF of the SLM-OFDM signal is represented as [27]

$$\begin{array}{c} Pr\left(PAPR>PAPR_{0}\right)\approx {\left(1-{\left(1-e^{-PAPR_{0}}\right)}^{\alpha N}\right)}^{V}. \end{array}$$ (17)

where *V* is the number of different phase sets. In this paper, we multiply only two kinds of phases, 1 or −1 and use a randomly generated phase sets. Of course, the number of elements in the phase set is the same as the number of data in one block or length of IFFT. The number of phase sets, *V* should be chosen carefully between performance and complexity trade-off. As *V* increases, the amount of the improvement of the PAPR reduction also increases. Simply increasing *V* causes high computational complexity and delay. Also, as increasing *V*, the performance improvement is rather limited. As observed from Fig 4, if *V* is larger than 32, there is only a slight performance improvement. This is also one of the motivations for using combined technique.

### Energy efficiency

In this subsection, we define the EE metric for the performance analysis. The EE is usually expressed as:

$$\begin{array}{c} EE=SE/P_{r}, \end{array}$$ (18)

where *SE* is the achievable rate in bit per second per Hz, and *P*_*r*_ is the related power consumption. For simplicity, we only consider PA power consumption, *P*_*pa*_ and VLSI power consumption for the computation of IFFT, *P*_*i*_. Then, the *P*_*r*_ can be divided into two parts as:

$$\begin{array}{c} P_{r}=P_{pa}+P_{i}, \end{array}$$ (19)

It is obvious that there are another power consumption components that can affect to the EE. However, the rest of power consumption components have little effect on the EE comparison of the proposed structure and conventional structure.

The relationship between TX power, *P*_*tx*_, and *P*_*pa*_ can be expressed as follows [30]:

$$\begin{array}{c} P_{tx}=\eta P_{pa}, \end{array}$$ (20)

where *η* is the PA efficiency. *η* can be changed depending on the PA type we use. i.e., Class-A, Class-B, Class-AB, Doherty PA and so on. Using different PA can affect the energy efficiency, but the design logic of combined PAPR reduction scheme is not changed. For example, in the case of class B PA, the *η* is defined as [30]:

$$\begin{array}{c} \eta \left(\%\right)=\frac{\pi }{4p}\times 100, \end{array}$$ (21)

where *p* is the square-root of input back-off (IBO), $\sqrt{IBO}$. IBO is the ratio of the maximum allowable input power or satuartion input power of PA, *P*_*max*_ and the average power of input signal *P*_*ave*_ [10, 30]:

$$\begin{array}{c} IBO\left(\text{dB}\right)=10{\text{log}}_{10}(\frac{P_{max}}{P_{ave}}), \end{array}$$ (22)

Increasing *IBO* reduces distortion, but also reduces power efficiency.

The *η* heavily depends on the PAPR of OFDM signal, and the PAPR of OFDM signal also depends on the system bandwidth and/or the number of subcarriers [30]. From Figs 1 and 5, we already witnessed that the PAPR of OFDM signal with no PAPR reduction is around 11.7dB from the 10^−3^ of the CCDF. For the computation of *P*_*i*_, first we need to define the Giga FLoating-point Operations Per Second (Gflop) for the IFFT which can be represented as [31–33]:

$$\begin{array}{ccc} \zeta (\text{Gflop}) & = & \frac{\left(T_{u}B\right)}{T_{s}}\cdot \log_{2}\left(T_{u}B\right), \end{array}$$ (23)

The system parameters are shown in Table 1. We took the parameters from the current 3GPP LTE system.

**Table 1:** System parameters.

Symbols	Description	Values
*B*	Bandwidth	10*MHz*
*T*_*sl*_	Slot length	0.5*ms*
*T*_*p*_	Pilot length in one slot	0.024*ms*
*T*_*s*_	Symbol duration	71.4*us*
*T*_*g*_	Guard Interval (GI)	4.7*us*
*T*_*u*_	Symbol without GI	66.7*us*
*T*_*d*_	Delay spread	4.7*us*

Then the *P*_*i*_ with SLM can be represented as,

$$\begin{array}{c} P_{i}=V\cdot \varepsilon \cdot \zeta . \end{array}$$ (24)

where *ε* is the VLSI efficiency which can be represented as Gflop/W. Here W represents the Watt. In this paper, we assume *ε* = 5Gflop/W.

Now, we should derive the achievable SE to get the EE metric in (18). The received signal at the receiver, *y*(*n*) can be represented as:

$$\begin{array}{ccc} y(n) & = & \sqrt{\rho }h\left(n\right)\hat{x}\left(n\right)+w\left(n\right)\\ & = & \sqrt{\rho }h\left(n\right)\alpha x\left(n\right)+\sqrt{\rho }h\left(n\right)d\left(n\right)+w\left(n\right). \end{array}$$ (25)

where *ρ* is the received power, *w*(*n*) is the Additive White Gaussian Noise (AWGN), and *h*(*n*) is the channel coefficient which can be decomposed as

$$\begin{array}{c} h(n)=g(n)\cdot \xi (n), \end{array}$$ (26)

where *g*(*n*) is the zero-mean and unit variance i.i.d. Rayleigh fading channel coefficient, and *ξ*(*n*) is the path loss component. Then from (26), we can derive the signal to noise and distortion ratio (SNDR) as follows:

$$\begin{array}{c} \gamma =\frac{\rho \xi^{2}\left(n\right)E\left[{\left|\alpha x\left(n\right)\right|}^{2}\right]}{\rho \xi^{2}\left(n\right)E\left[{\left|d\left(n\right)\right|}^{2}\right]+E\left({\left|w\left(n\right)\right|}^{2}\right)} \end{array}$$ (27)

The approximated achievable rate can be represented as:

$$\begin{array}{c} SE\approx \kappa \cdot E[\log_{2}(1+\gamma )], \end{array}$$ (28)

where $\kappa =(\frac{T_{sl}-T_{p}}{T_{sl}})\cdot (\frac{T_{u}}{T_{s}})$ is the scaling factor for the pilot overhead and guard interval. In this paper, we assume the pilot overhead is 4.8% from 3GPP LTE system.

Finally, EE can be defined as

$$\begin{array}{c} EE=\frac{\kappa \cdot E[\log_{2}(1+\gamma )]}{\left(P_{pa}+P_{i}\right)}. \end{array}$$ (29)

We will use (29) as the metric for the performance analysis.

## Design methodology for the combination of SLM and clipping

In this section, we show the energy efficient design methodology using the combination of SLM and clipping technique. As mentioned, the combination of SLM and clipping technique could improve EE performance. We recommend placing the SLM before clipping, because it can reduce clipping distortion and peak re-growth after filtering. Combination of SLM and clipping technologies were already discussed in several literatures [13, 14]. However, there is no literature to introduce the design methodology and show the EE gain of the combined scheme.

There are several benefits by using clipping and SLM together. That is, we can choose between two policies that minimize distortion and minimize power consumption. If there is a target PAPR that signal can pass safely through the nonlinear devices such as PA and digital-to-analog converter (DAC), we should choose appropriate *V* and CR, *ν* for the target PAPR. To minimize distortion, we should use high *V* and high CR, *ν*. Conversely, to minimize power consumption, we should choose low *V* and low CR, *ν*. Also out-of-band radiation can be significantly reduced by using the combined technique. It is another benefit that we can choose EE/SE performance—complexity trade-off.

**Fig 6:**
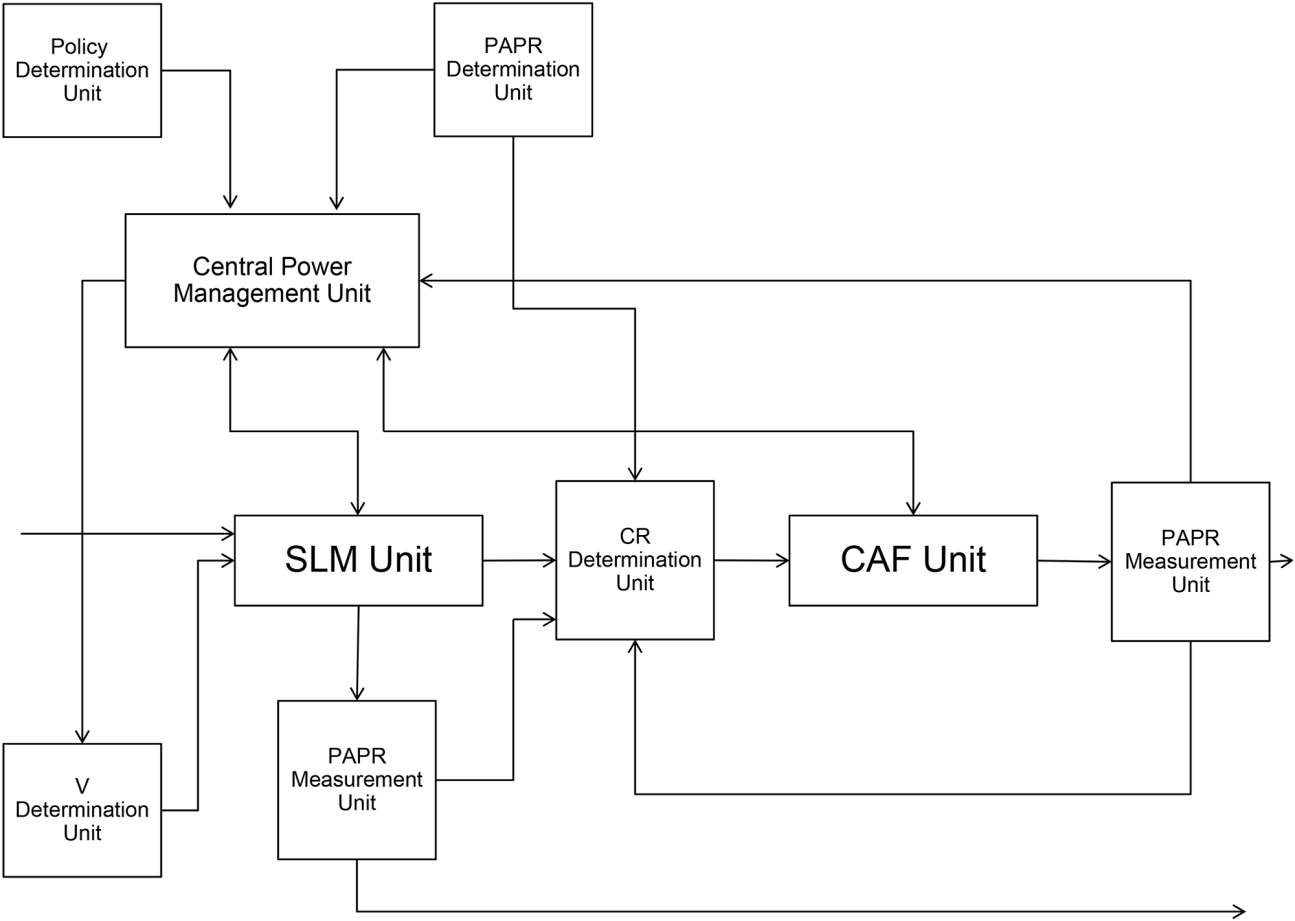
Proposed PAPR reduction apparatus that uses SLM and clipping technique.

**Fig 7:**
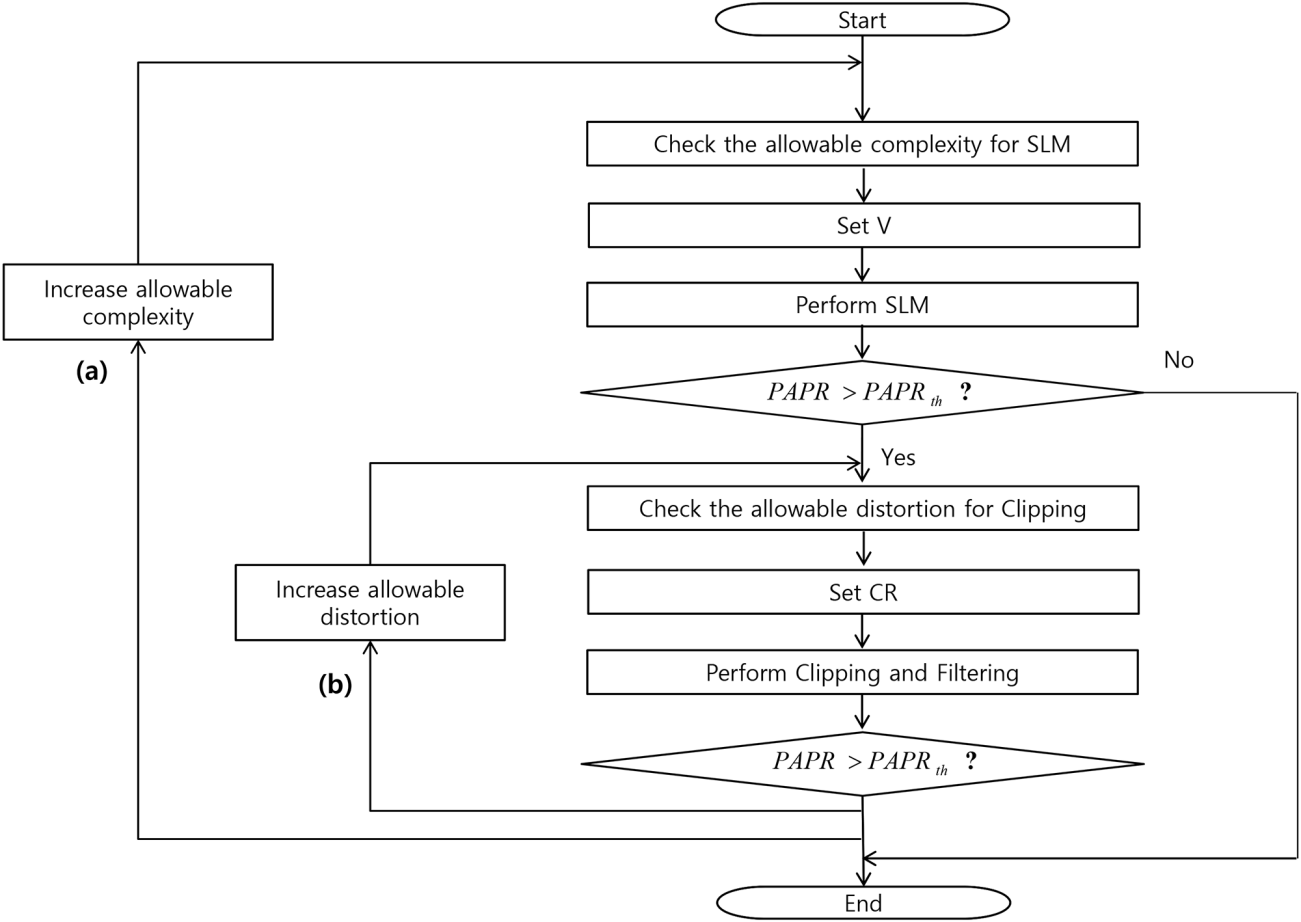
Flow chart for an example of using combination of SLM and clipping PAPR reduction technique.

Fig 6 shows proposed PAPR reduction apparatus that uses SLM and clipping technique. The policy determination unit determines whether the system will pursuit distortion minimization or power consumption minimization. Based on the information, central power management unit determines the allowable power and computational delay to be used for the SLM technique. Usually the power consumption of CAF is almost fixed if the iterative technique is not used. Acceptable PAPR should also be determined prior to the PAPR reduction process based on the nonlinear circuits conditions and/or expected system performance. After the number of phase sets *V* is determined based on the allowable power consumption and computational delay for SLM, the SLM PAPR reduction technique is performed. Based on the PAPR of OFDM signal after SLM, CR is determined and CAF is performed. If the measured PAPR is within an acceptable range, the signal goes out of the PAPR reduction apparatus. Otherwise, based on the policy, the PAPR measurement result is returned to the central power management or CR determination unit. For the distortion minimization policy, it goes to the central power management unit. For the power consumption minimization policy, it goes to the CR determination unit. Also, if the signal is within the acceptable range after the SLM technique, it is possible for the signal to escape from the PAPR reduction apparatus after the SLM technique. The process based on the PAPR reduction apparatus is depicted as a flow chart in Fig 7. In Fig 7, (a) indicates PAPR reduction process according to the distortion minimization policy, while (b) indicates the PAPR reduction process according to power consumption minimization policy. After enough operation, look-up table (LUT) can be filled which can be located in central power management unit. Then, once the allowable PAPR and policy are given, the apparatus can refer the LUT and choose the best *V* and *CR*, *ν* in a given policy, PAPR, and other situations. The LUT should be updated continuously after pre-defined interval.

For the better understand and implementation, we provide the detailed algorithm as pseudo-code type in Algorithm 1.

**Algorithm 1**: Combination of SLM and Clipping.

**1** Check the allowable complexity for SLM;

**2** Set *V*;

**3** Perform SLM;

**4** **if** *PAPR* > *PAPR*_0_;

**5** **then**

**6**  Check the allowable distortion for clipping;

**7**  Set CR;

**8**  Perform CAF;

**9**  **if** (*PAPR* > *PAPR*_0_) & (Policy ← *min*{*Complexity*});

**10**  **then**

**11**   Increase allowable distortion;

**12**   Go to 6;

**13**  **else if** (*PAPR* > *PAPR*_0_) & (Policy ← *min*{*Distortion*});

**14**  **then**

**15**   Increase allowable complexity;

**16**   Go to 1;

**17**  **else**

**18**   Go to 21;

**19** **else**

**20**  Go to 21;

**21** Send the processed signal as output;

## Numerical results

In this section, we show the EE gain of the combined technique based on simulation and analytic results.

**Fig 8:**
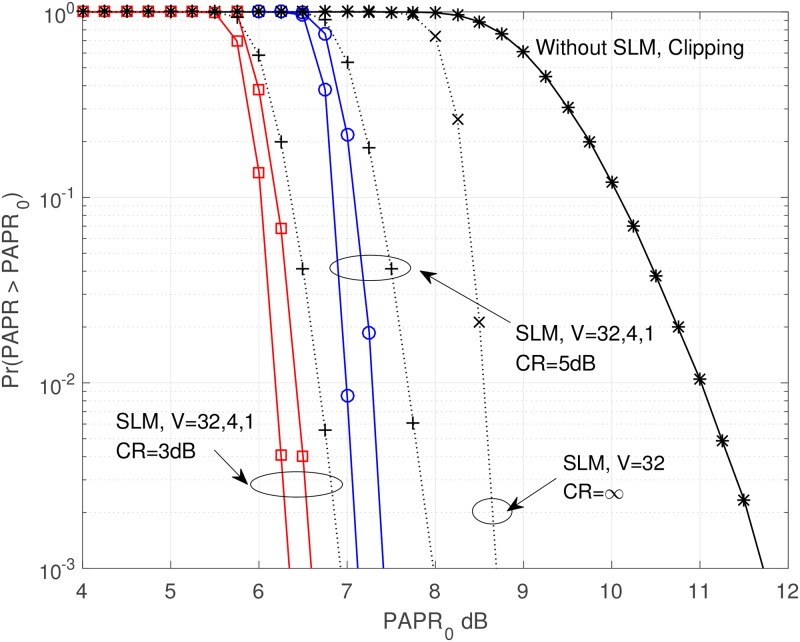
CCDF of OFDM signal using combined technique.

We present the PAPR performances of combined technique, clipping technique and SLM technique in Fig 8 using CCDF.

The solid lines of Fig 8 represent the CCDF of combined technique. The dotted lines represent that of clipping and/or SLM techniques for comparison purpose. It is obvious that for the PAPR reduction perspective, the combined technique gives much better PAPR performance than clipping and/or SLM technique. The PAPR performance when *CCDF* = 10^−3^ is summarized in Table 2. The PAPR performance improvement when using combined technique compared to using only clipping technique is around 10.7% (7.961 → 7.109dB) in the case of *CR* = 5dB, and 8.38% (6.907 → 6.328dB) in the case of *CR* = 3dB. Also the PAPR performance improvement when using combined technique compared to using only SLM technique is around 18.15% (8.685 → 7.109dB) in the case of *CR* = 5dB, and 27.14% (8.685 → 6.328dB) in the case of *CR* = 3dB.

**Table 2:** PAPR performance (dB) when *CCDF* = 10^−3^.

	*CR* = ∞	*CR* = 5dB	*CR* = 3dB
*V* = 1	11.7	7.961	6.907
*V* = 4	9.825	7.409	6.581
*V* = 32	8.685	7.109	6.328
*V* = 64	8.456	-	-

**Fig 9:**
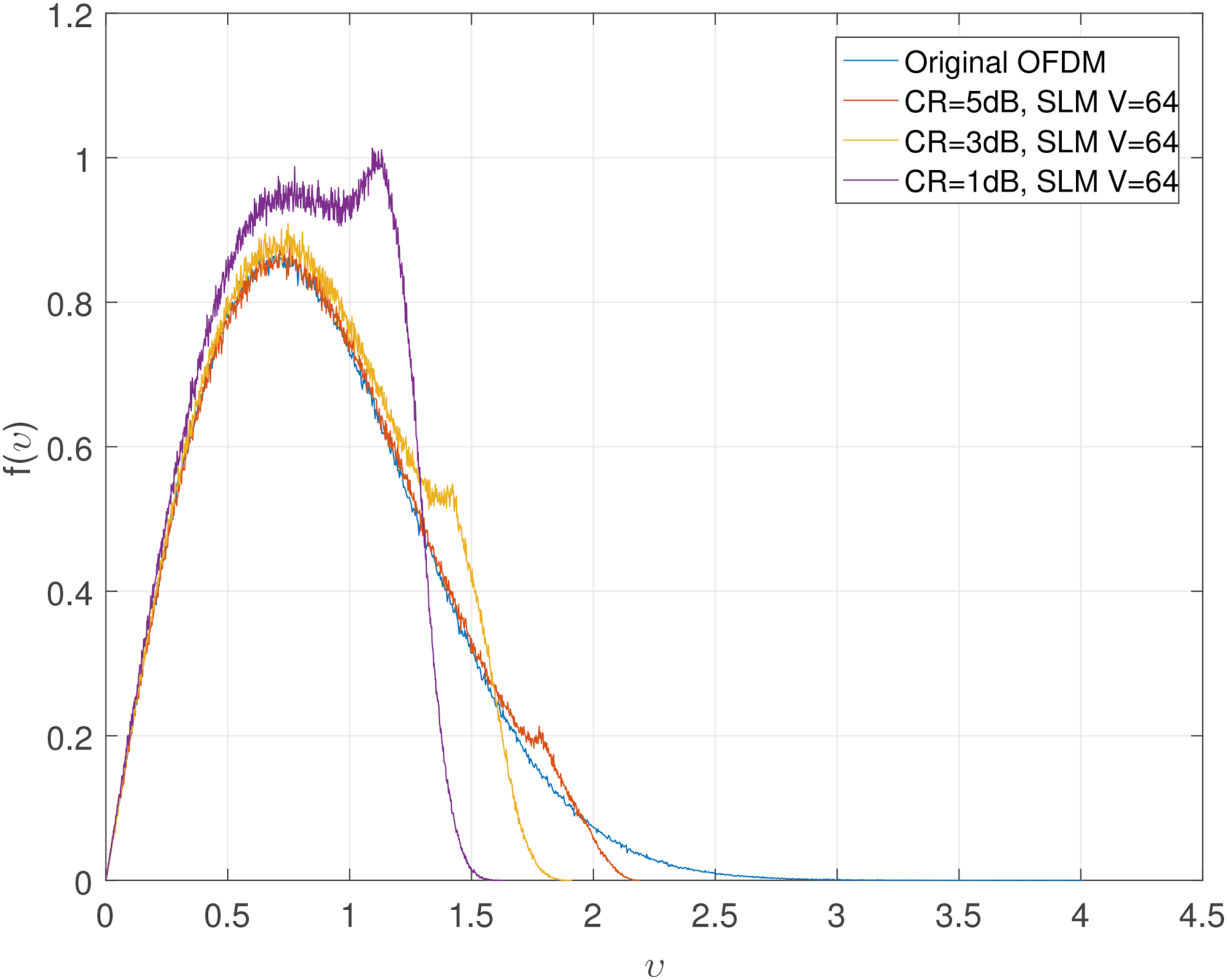
PDF of OFDM signal amplitude using combined technique.

The clipping distortion is inevitable even we use the combined technique as shown in Fig 9. The amplitude distribution characteristic of combined technique is similar with that of only clipping due to the clipping distortion. However, the distortion could be much less than using only clipping technique, because the PAPR of the OFDM signal is reduced by using SLM before passing through the clipping process.

**Fig 10:**
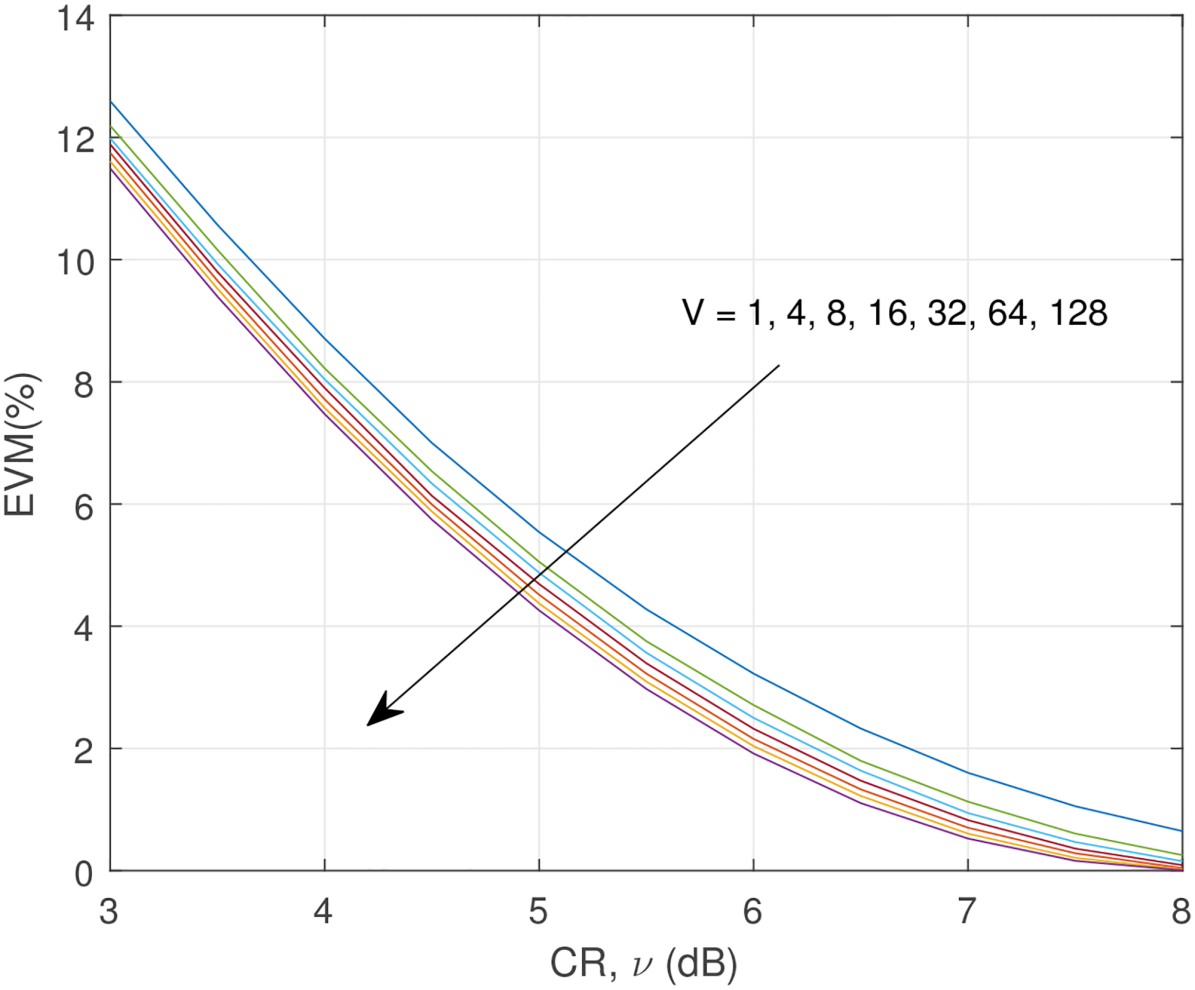
Error vector magnitude (EVM) (%).

**Fig 11:**
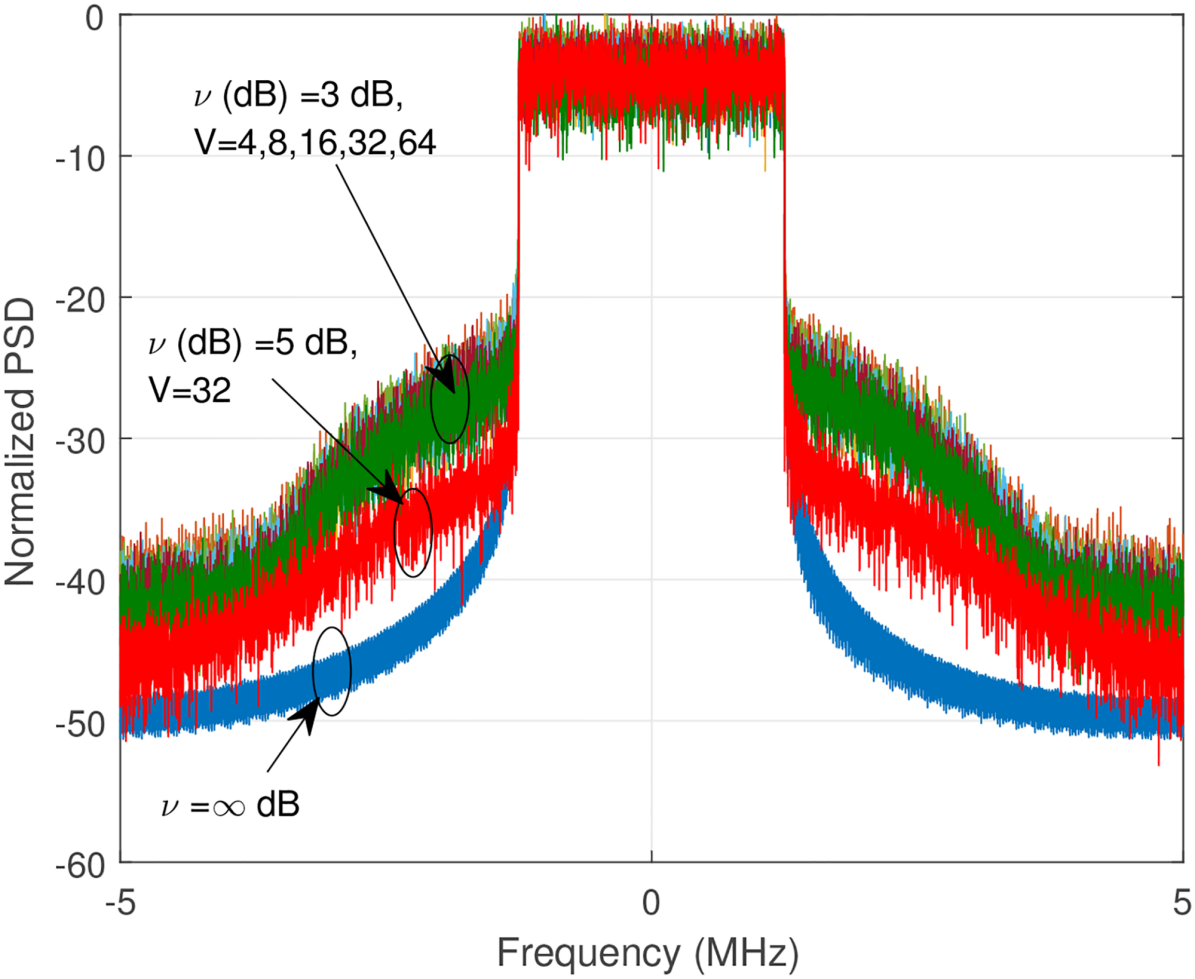
Normalized power spectral density (PSD).

Let us show the error vector magnitude (EVM), and out-of-band power radiation (OOBPR) analysis using normalized power spectral density (PSD) in Figs 10 and 11. EVM is a measure used to quantify how accurately the signal is transmitted, and defined as the ratio of the power of error vector to the power of reference, and is expressed as follows [34]:

$$\begin{array}{c} \text{EVM}\left(\%\right)=\sqrt{\frac{\sum_{n}|x\left(n\right)-\hat{x}\left(n\right){|}^{2}}{\sum_{n}|x\left(n\right){|}^{2}}}\times 100, \end{array}$$ (30)

When *ν* = 5dB, without using SLM, EVM is 5.54%. It is reduced to 4.37% if we combine with SLM, V = 64, thus we can get around 26.77% EVM improvement using combined technique. Regarding OOBPR, SLM can reduce the OOBPR, but the improvement is not so noticeable. Increasing CR is a good choice to reduce the OOBPR.

Even though the combined technique shows better performance, the complexity is higher than using only clipping and/or SLM. For the fair comparison, now we show the EE comparison using the metric we defined previously. EE metric can reflect the complexity in the denominator.

**Fig 12:**
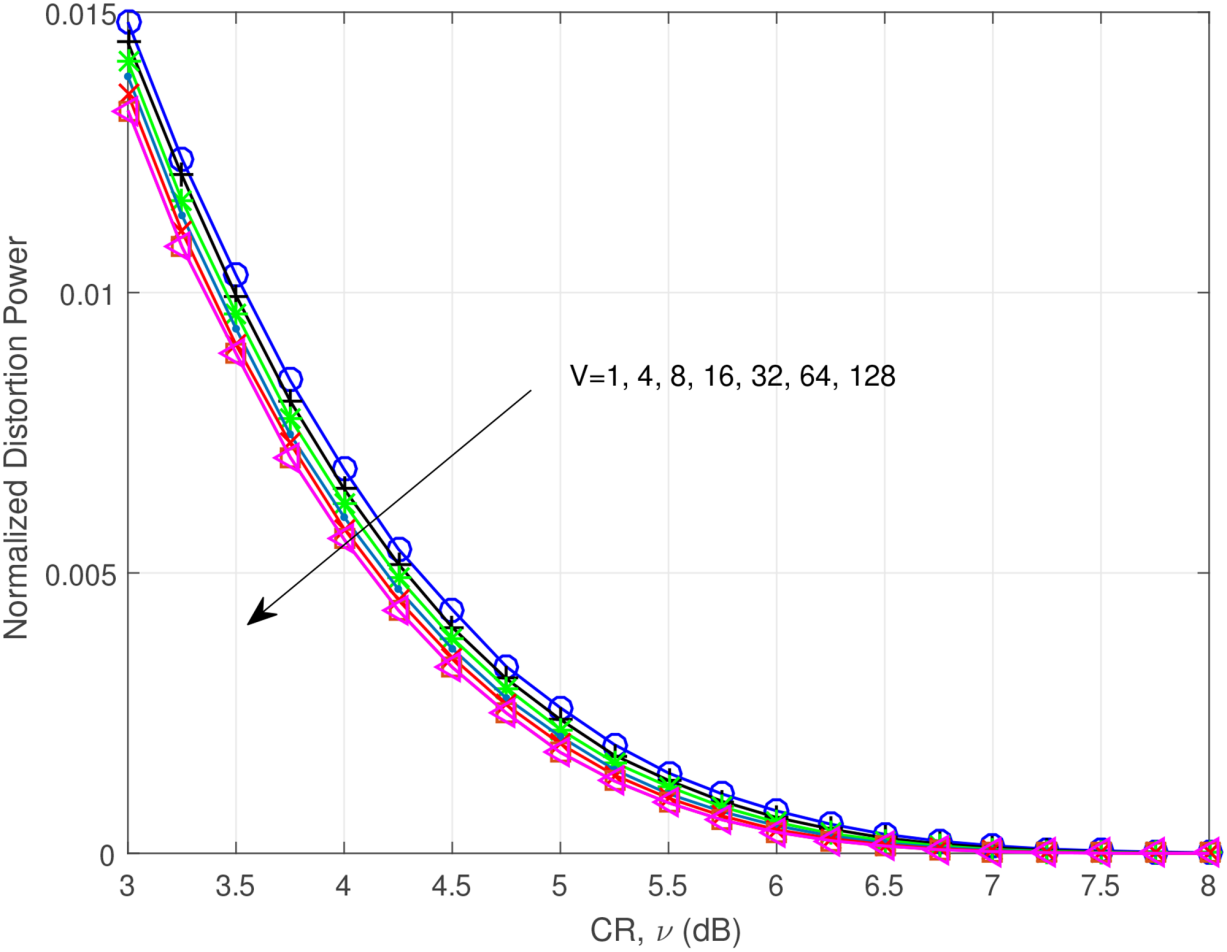
Normalized distortion power versus CR, V = 1, 4, 8, 16, 32, 64, 128.

First, for the analytical results, we investigate the distortion power of the combined technique. Fig 12 presents the normalized distortion power versus CR. Here the normalized distortion power indicates the distortion power when the transmission power is normalized to unity. As observed, it is obvious that as *V* increases, the distortion power reduces.

**Fig 13:**
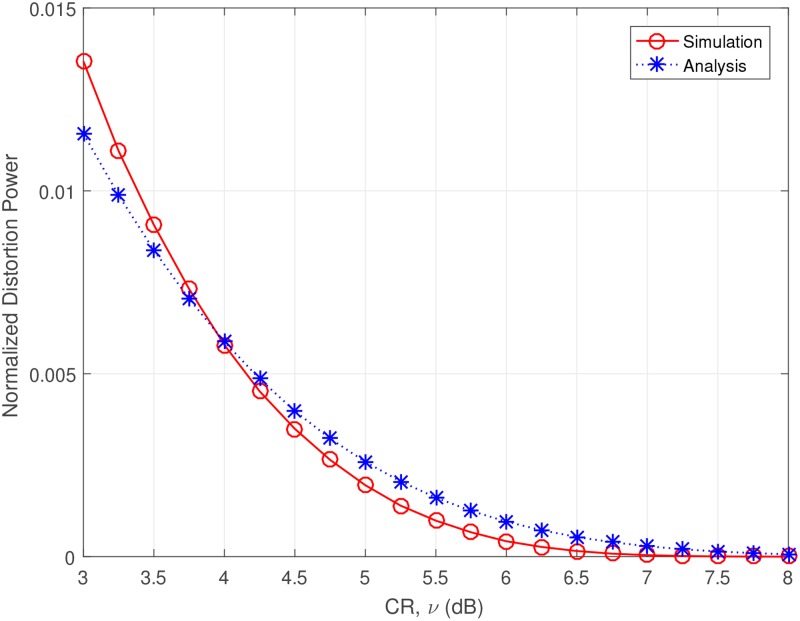
Normalized distortion power versus CR, matching analysis to the simulaiton, V = 32.

The Eq (16) is only valid for the signal which does not use oversampling and combination with SLM. Without oversampling, the clipping technique is meaningless in real situation. We present the simplified empirical model of the distortion power for the clipping and SLM combination technique with oversampling, which can be represented as,

$$\begin{array}{c} E\left({\left|d_{c}\left(n\right)\right|}^{2}\right)=\chi \cdot \left(e^{-\nu^{2}}-\sqrt{\pi }\nu \cdot erfc\left(\nu \right)\right). \end{array}$$ (31)

where *χ* is the adjustable factor for the distortion power of the combined technique. We show the comparison graph in Fig 13. We chose *V* = 32 and *χ* = 0.54. *χ* = 0.54 is chosen based on the empirical distortion power modeling of the combination of oversampling, clipping and SLM. Clipping with SLM is highly nonlinear process, thus it is very difficult to derive the theoretical model of distortion power. Based on searching and curve fitting methods, we can find the adjustable factor of (31) and complete the model. As we can see, Eq (31) matches well with the simulation result, so we will use it for the analytic result.

**Fig 14:**
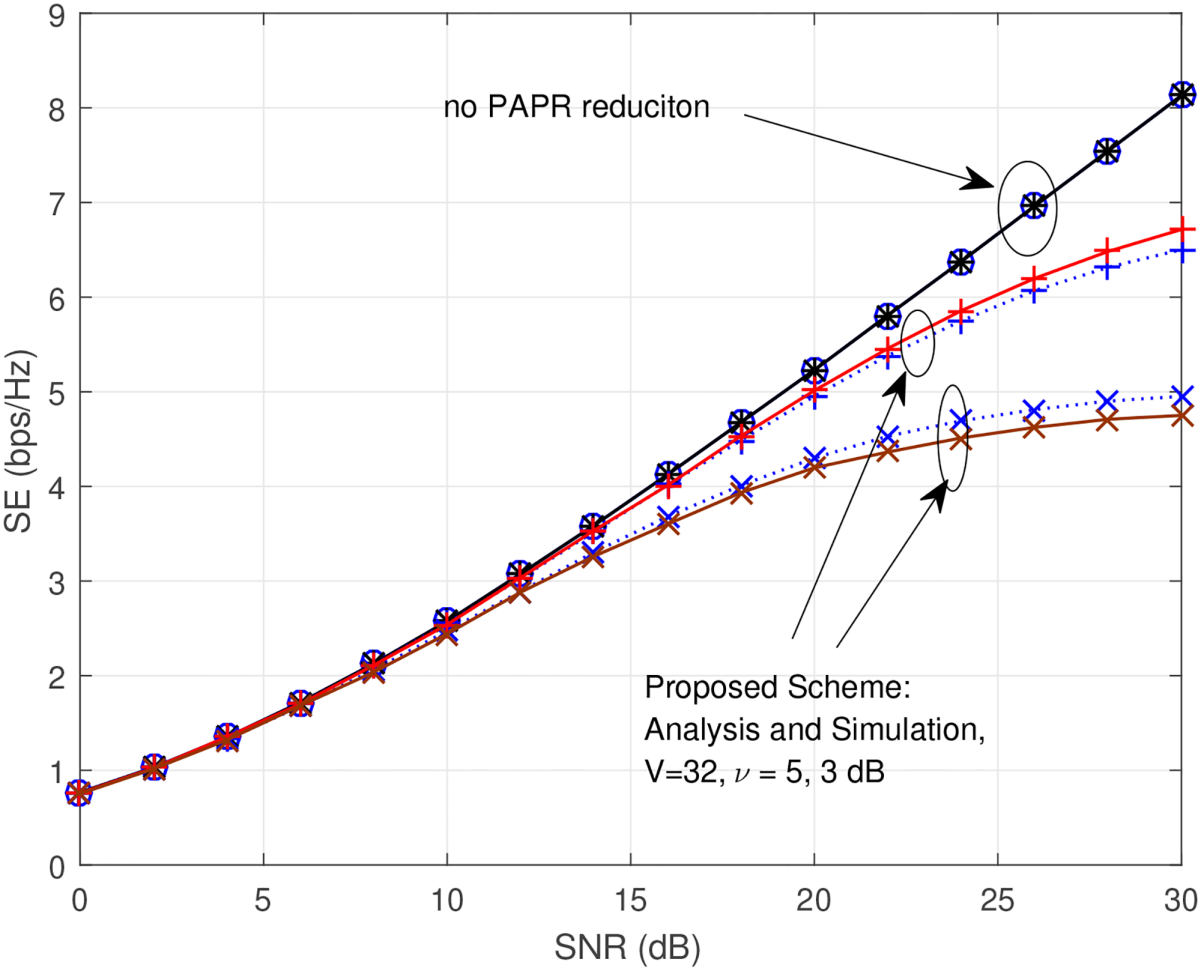
SE (bps/Hz) versus SNR (dB), when *V* = 32, *ν* = ∞, 5, 3*dB*. Solid lines indicate the simulation results, and dotted line indicates the anlaytic results.

**Fig 15:**
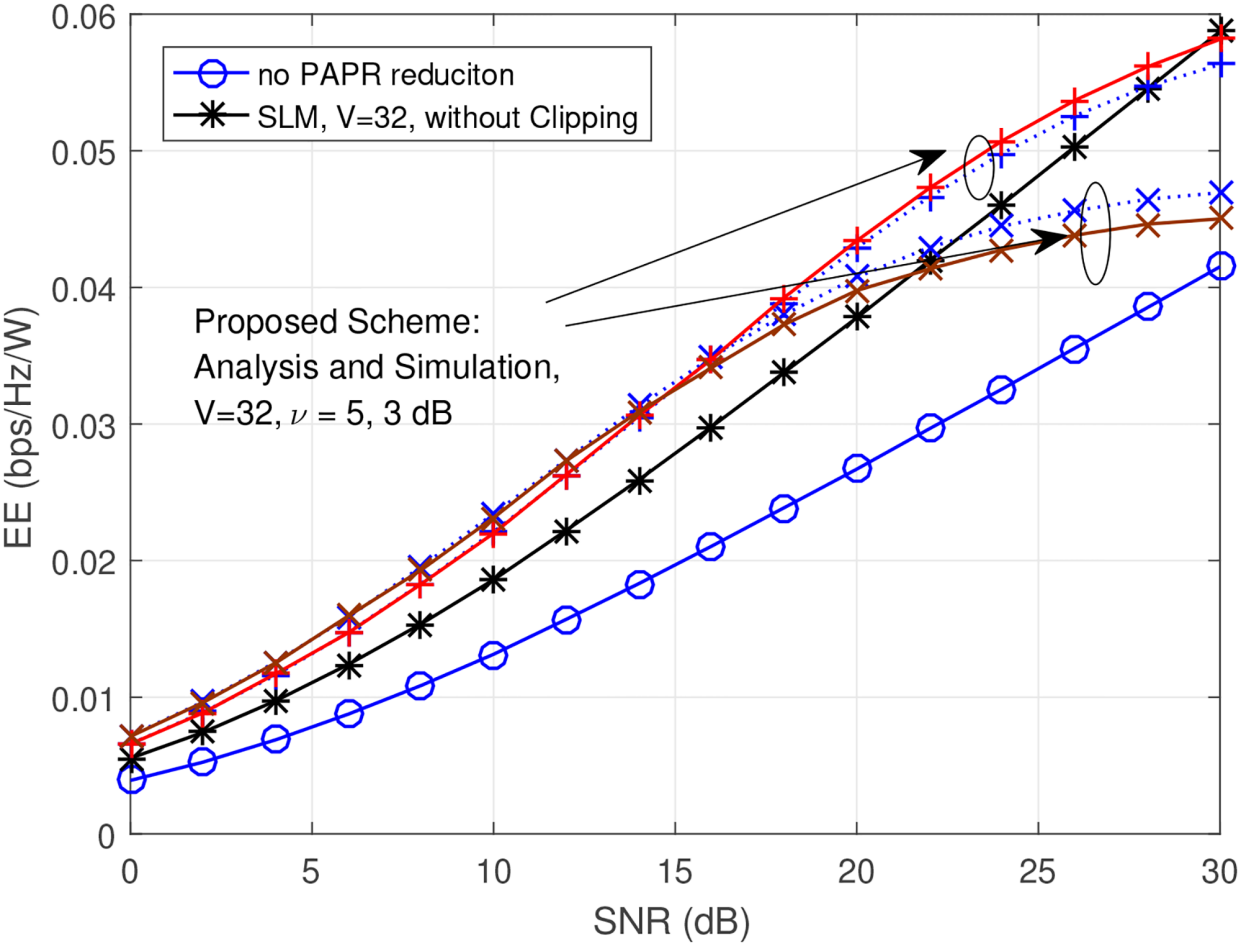
EE (bps/Hz) versus SNR (dB), when *V* = 32, *ν* = ∞, 5, 3dB. Solid lines indicate the simulation results, and dotted lines indicate the anlaytical results.

Now let us show the SE (bps/Hz) versus SNR (dB) in Fig 14. For the parameters of the combined technique, we used *V* = 32, *ν* = ∞, 5, 3dB. Solid lines indicate the simulation results, and dotted lines indicate the analytic results. As observed in Fig 14, the analytic results are well-matched with the simulation results. In the case of clipping with SLM, as clipping amount increases, the SE reduces due to the clipping noise. In the case of no PAPR reduction shows the best SE since it does not have any signal distortion. In real system, in order to achieve the same SNR in the case of no PAPR reduction and in the case of clipping with SLM, the former must use expensive devices to emit more power than the latter. For example, SNR for the case of no PAPR reduction can be represented as

$$\begin{array}{c} {\text{SNR}}^{\text{no}}=P_{tx}^{\text{no}}\cdot \frac{1}{N_{0}B}=\frac{P_{pa}^{\text{no}}}{\eta^{\text{no}}}\cdot \frac{1}{N_{0}B} \end{array}$$ (32)

where $P_{tx}^{\text{no}}$ is the TX power, $P_{pa}^{\text{no}}$ is the PA power consumption, *η*^no^ is the PA efficiency for the case of no PAPR reduction, and *N*_0_*B* is the noise power in a given bandwidth. Then, SNR for the case of proposed scheme can be represented as

$$\begin{array}{c} {\text{SNR}}^{p}=P_{tx}^{p}\cdot \frac{1}{N_{0}B}=\frac{P_{pa}^{p}}{\eta^{p}}\cdot \frac{1}{N_{0}B} \end{array}$$ (33)

where $P_{tx}^{p}$ is the TX power, $P_{pa}^{p}$ is the PA power consumption, *η*^p^ is the PA efficiency for the case of proposed scheme. For the case of SNR^no^ = SNR^p^, it is obvious that $P_{pa}^{\text{no}}$ must be greater than $P_{pa}^{p}$ (i.e., $P_{pa}^{\text{no}}>P_{pa}^{p}$) because the efficiency of the case of no PAPR reduction is smaller than the efficiency of the case of proposed scheme (*η*^no^ < *η*^p^). These factors are reflected in the denominator of EE. Also, as we mentioned previously, the complexity of the algorithm is reflected in the denominator of EE. For these reasons, we present the EE comparison in Fig 15 for more fair comparison. We applied appropriate input back-off (IBO) based on PAPR reduction performance. The IBO criterion is when *CCDF* = 10^−3^. In the low SNR region, the case of *ν* = 3dB shows the best performance. If SNR becomes higher than 15dB and less than 30dB, the case of *ν* = 5dB shows the best performance. If SNR is very high which is higher than 30dB, only SLM technique shows the best performance. The clipping with SLM and/or SLM techniques shows better EE than the case of no PAPR reduction for all of the SNR region.

**Fig 16:**
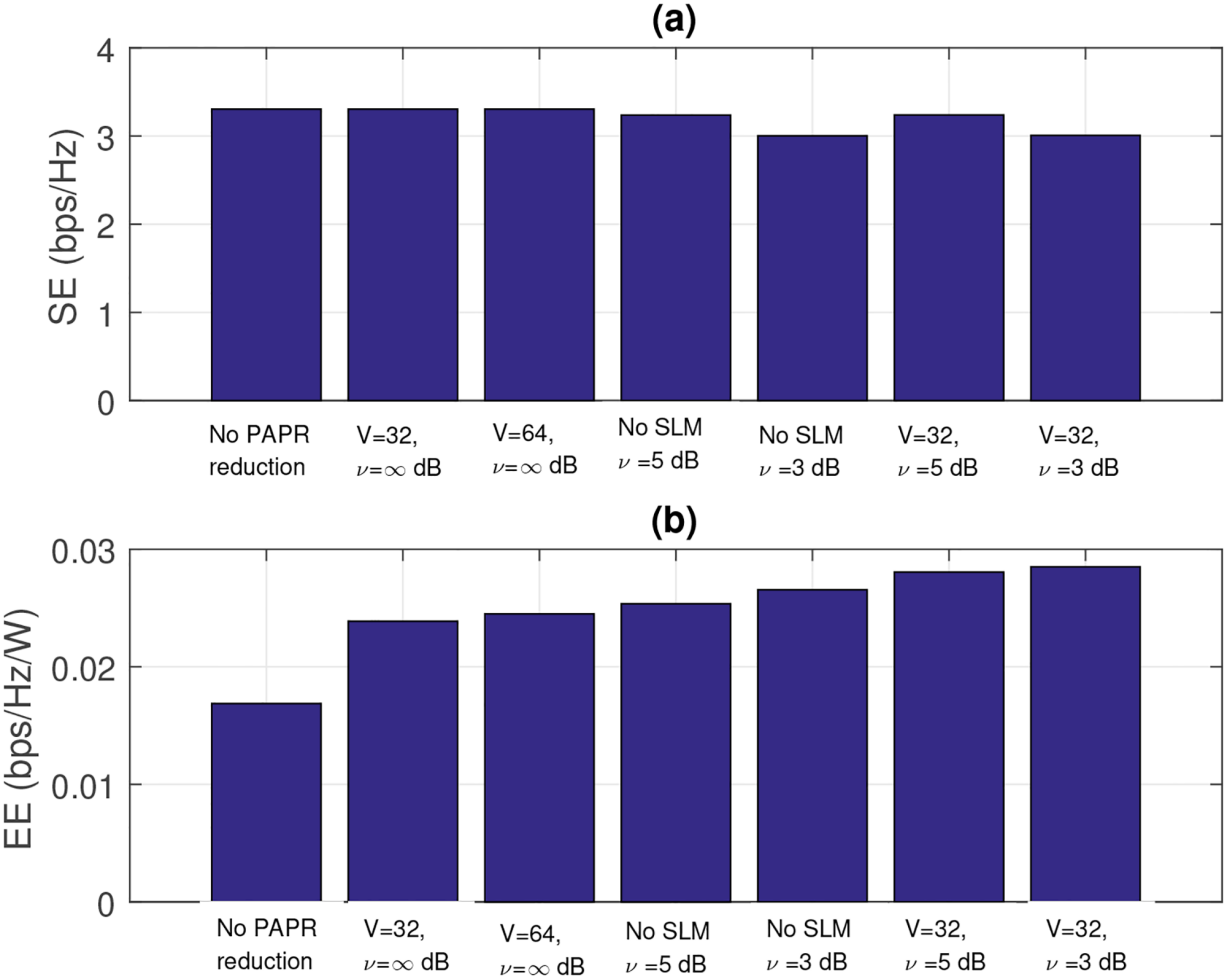
(a) Cell SE (bps/H) and (b) Cell EE (bps/Hz/W).

As a last analysis, we present simulation results of cell SE and EE in Fig 16. For the numerical simulations, we use a macro-cell type setup with 2GHz carrier frequency. The path loss in dB is modeled as 128.1 + 37.6*log*(Υ) with distance Υ in kilometers [35]. We assume a cell radius of 2000 meters with a cell-hole radius of 100 meters. The user locations are uniform-random, and an average was taken over 10,000,000 trials.

We can see from (a) of Fig 16, as CR increases, SE decreases, but when clipping and SLM are combined, the decrease is smaller. Also as observed from (b) of Fig 16, as *V* and/or CR increases, EE increases, and the increase is more dominant when we use the combined technique.

The EE increments can be expressed as relative EEs. We define the relative EE, *EE*_*r*_ as follows:

$$\begin{array}{c} EE_{r}=EE/EE_{ref} \end{array}$$ (34)

**Fig 17:**
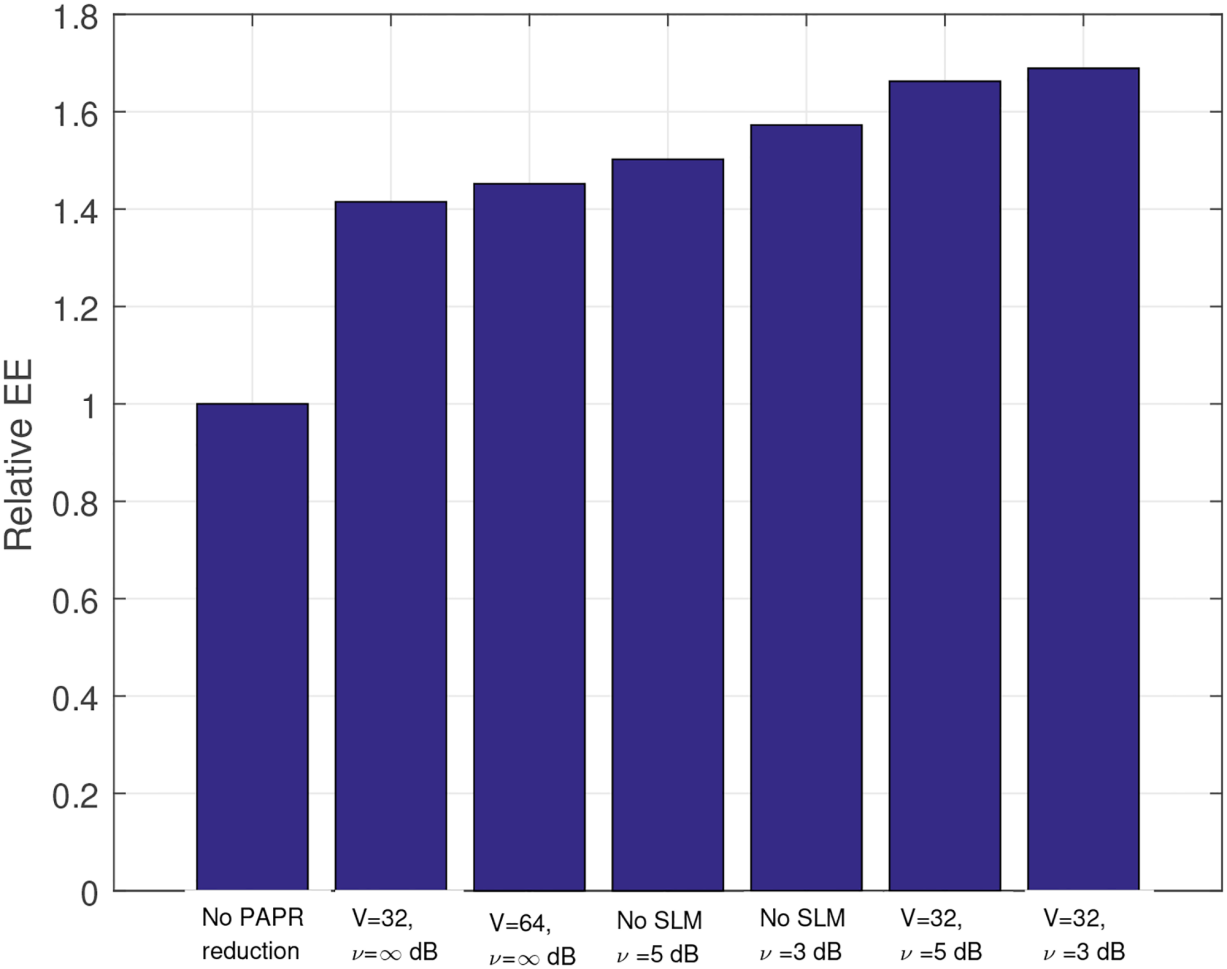
Relative EE comparison between single technique and combined technique.

*EE*_*ref*_ indicates the EE when no PAPR reduction technique is applied. Fig 17 present the relative EE for the cases of clipping only, SLM only, and combined technique. When we apply SLM with *V* = 32, the relative EE increases up to 1.415. Even though *V* increases to 64, the increase in EE is marginal (2.47% (1.415 → 1.45)). In the case of clipping, when *ν* = 5dB, the relative EE is 1.5. Even though *ν* reduces to 3dB, the increase in relative EE is also marginal (4.67% (1.5 → 1.57)). By using the combined technique, the relative EE reaches 1.69, when *V* = 32, *ν* = 3dB. The increment for using only SLM with *V* = 32 is 19.34% (1.415 → 1.69), and the increment for using only clipping with *ν* = 3dB is 7.64% (1.57 → 1.69). As shown in the analytical and simulation results, the combined technique can achieve much higher EE than the single technique.

## Conclusions

In this paper, we showed the methodology and analysis for the combination of two PAPR reduction techniques, clipping and SLM. By combing the two PAPR reduction techniques, we witnessed that we can get much higher EE gain than only using single technique. SLM should be located before clipping to minimize the distortion. We can set the policies based on system requirements, minimize complexity or minimize distortion. The analytical distortion power is also derived which is well-matched with simulation results. We showed that the proposed technique can increase EE by 69% compared to no PAPR reduction, and by 19.34% compared to only using SLM technique. The combined technique can be used for any kind of transmission system which uses high PAPR signal, but needs high EE.

## References

[1] Lee BM, Kim Y (2016) Design of an Energy Efficient Future Base Station with Large-Scale Antenna System. Energies, 9 (12), 1083 10.3390/en9121083

[2] Tham M-L, Chow C-O, Xu Y-H, Ramli N (2016) Two-Level Scheduling for Video Transmission over Downlink OFDMA Networks. PLoS ONE 11(2): e0148625 10.1371/journal.pone.0148625 26906398 PMC4764368

[3] Shamsan ZA, Al-hetar AM (2016) An Improved Mathematical Scheme for LTE-Advanced Coexistence with FM Broadcasting Service. PLoS ONE 11(11): e0136912 10.1371/journal.pone.0136912 27855216 PMC5113892

[4] Uthirajoo E, Ramiah H, Kanesan J, Reza AW (2014) Wideband LTE Power Amplifier with Integrated Novel Analog Pre-Distorter Linearizer for Mobile Wireless Communications. PLoS ONE 9(7): e101862 10.1371/journal.pone.0101862 25033049 PMC4102479

[5] Peng J, He S, Wang B, Dai Z, Pang J (2016) Digital Predistortion for Power Amplifier Based on Sparse Bayesian Learning. IEEE Trans. Circuits Syst. II, Exp. Briefs, 63 (9), 828–832. 10.1109/TCSII.2016.2534718

[6] Zhao J, Liu Y, Yu C, Yu J, Li S (2016) A Modified Band-Limited Digital Predistortion Technique for Broadband Power Amplifiers. IEEE Commun. Lett., 20 (9), 1800–1803. 10.1109/LCOMM.2016.2585652

[7] Lee BM, de Figueiredo RJP (2006) Adaptive Pre-Distorters for Linearization of High Power Amplifiers in OFDM Wireless Communications. Circuits, Systems & Signal Processing, Birkhauser Boston, 25 (1), 59–80. 10.1007/s00034-004-0901-x

[8] Jiang T, Wu Y (2008) An Overview: Peak-to-Average Power Ratio Reduction Techniques for OFDM Signals. IEEE Trans. Broadcast., 54 (2), 257–268. 10.1109/TBC.2008.915770

[9] Elhelw AM, Badran EF (2015) Semi-Blind Error Resilient SLM for PAPR Reduction in OFDM Using Spread Spectrum Codes. PLoS ONE 10(5): e0127639 10.1371/journal.pone.0127639 26018504 PMC4446332

[10] Jiang T, Li C, Ni C (2013) Effect of PAPR Reduction on Spectrum and Energy Efficiencies in OFDM Systems with Class-A HPA Over AWGN Channel. IEEE Trans. Broadcast., 59 (3), 513–519. 10.1109/TBC.2013.2253814

[11] Jiang T, Zhu G (2005) Complement Block Coding for Reduction in Peak-to-Average Power Ratio of OFDM Signals. IEEE Commun. Mag., 43 (9), S17–S22. 10.1109/MCOM.2005.1509967

[12] Jiang T, Guizani M, Chen HH, Xiang W, Wu Y (2008) Derivation of PAPR Distribution for OFDM Wireless Systems Based on Extreme Value Theory, IEEE Trans. Wireless Commun., 7 (4), 1298–1305.

[13] Ochiai H, Imai H (2000) Performance of the deliberate clipping with adaptive symbol selection for strictly band-limited OFDM systems. IEEE J. Select. Areas Commun., 18, 2270–2277. 10.1109/49.895032

[14] Lee BM, Kim Y, de Figueiredo RJP (2012) Performance Analysis of the Clipping Scheme with SLM Technique for PAPR Reduction of OFDM Signals in Fading Channels. Wireless Personal Communications, 63 (2), 331–344. 10.1007/s11277-010-0136-z

[15] Yoshizawa R, Ochiai H (2015) Effect of Clipping and Filtering with Distortionless PAPR Reduction for OFDM Systems, IEEE VTC-Fall.

[16] Wang L, Tellambura C (2005) A Simplified Clipping and Filtering Technique for PAR Reduction in OFDM Systems. IEEE Signal Process. Lett., 12 (6), 453–456. 10.1109/LSP.2005.847886

[17] Armstrong J (2002) Peak-to-average power reduction for OFDM by repeated clipping and frequency domain filtering. Electron. Lett., 38, 246–247. 10.1049/el:20020175

[18] van Nee R, de Wild A (1998) Reducing the peak-to-average power ratio of OFDM. in Proc. IEEE Vehicular Technology Conf. (VTC98), 2072–2076.

[19] Ochiai H, Imai H (2001) On the distribution of the peak-to-average power ratio in OFDM signals. IEEE Trans. Commuin., 49 (2), 282–289. 10.1109/26.905885

[20] Wei S, Goeckel DL, Kelly PE (2002) A modern extreme value theory approach to calculating the distribution of the peak-to-average power ratio in OFDM systems. in Proc. IEEE International Conference on Communications, 3, 1686–1690.

[21] Li X, Cimini LJ Jr (1998) Effects of clipping and filtering on the performance of OFDM. IEEE Commun. Lett., 2 (5), 131–133. 10.1109/4234.673657

[22] Ochiai H, Imai H (2002) Performance analysis of deliberatly clipped OFDM singals. IEEE Trans. Commuin., 50, 89–101. 10.1109/26.975762

[23] Chen H, Haimovich AM (2003) Iterative estimation and cancellation of clipping noise for OFDM signals. IEEE Commun. Lett., 7 (7), 305–307. 10.1109/LCOMM.2003.814720

[24] Bussgang JJ (1952) Crosscorrelation functions of amplitude distorted Gaussian signals. Research Lab. Electron.

[25] Lee BM, Kim Y (2013) An adaptive clipping and filtering technique for PAPR reduction of OFDM signals, Circuits, Systems, & Signal Processing, 32 (3), 1335–1349. 10.1007/s00034-012-9512-0

[26] Bauml RW, Fischer RFH, Huber JB (1996) Reducing the peak-to-average power ratio of multicarrier modulation by selected mapping. Electronics Letters, 32, 2056–2057. 10.1049/el:19961384

[27] Muller SH, Huber HB (1997) OFDM with reduced peak-to-mean power ratio by optimum combination of partial transmit sequences. Electronics Letters, 33, 368–369. 10.1049/el:19970266

[28] Jayalath ADS, Tellambura C (2002) A blind SLM receiver for PAR-reduced OFDM. Proc. IEEE Vehicular Technology Conference, 219–222.

[29] Zhou TG, Baxley RJ, Chen N (2004) Selected mapping with monomial phase phase rotations for peak-to-average power ratio reduction in OFDM, Proc. Intl. Conf. on Communications, Circuits and Systems, 66–70.

[30] Cripps SC (2006) RF power amplifiers for Wireless Communications. second edition, Artech house.

[31] Yang H, Marzetta T (2013) Performance of Conjugate and Zero-Forcing Beamforming in Large-Scale Antenna Systems. IEEE Journal of Selected Areas in Comm., 31 (2), 172–179. 10.1109/JSAC.2013.130206

[32] Yang H, Marzetta T (2013) Total energy efficiency of cellular large scale antenna system multiple access mobile networks. IEEE Online Conference on Green Communications (GreenCom), 27–32.

[33] Yang H, Marzetta T (2015) Energy efficient design of massive MIMO: How many antennas?. 2015 IEEE 81st Vehicular Technology Conference (VTC Spring), 1–5.

[34] 3rd Generation Partnership Project (2017) Technical Specification Group Radio Access Network; Evolved Universal Terrestrial Radio Access (E-UTRA); User Equipment (UE) radio transmission and reception.

[35] ETSI (2012) Technical Report 136.931 Radio frequency (RF) Requirements for LTE Pico Node B, Tech. Rep.

